# Optimization of culture conditions for porcine corneal endothelial cells

**Published:** 2007-04-03

**Authors:** Stéphanie Proulx, Jean-Michel Bourget, Nicolas Gagnon, Sophie Martel, Alexandre Deschambeault, Patrick Carrier, Claude J. Giasson, François A. Auger, Isabelle Brunette, Lucie Germain

**Affiliations:** 1 Laboratoire d'Organogénèse Experiméntale (LOEX), Hôpital du St-Sacrement du Centre Hospitalier Affilié Universitaire de Québec and Department of Oto-Rhino-Laryngology and Ophthalmology, Université Laval, Québec, Canada; 2 School of Optometry, Université de Montréal, Montréal, Québec, Canada; 3 Ophthalmology Research Unit, Department of Ophthalmology, Maisonneuve Rosemont Hospital, Université de Montréal, Montréal, Québec, Canada

## Abstract

**Purpose:**

To optimize the growth condition of porcine corneal endothelial cells (PCEC), we evaluated the effect of coculturing with a feeder layer (irradiated 3T3 fibroblasts) with the addition of various exogenous factors, such as epidermal growth factor (EGF), nerve growth factor (NGF), bovine pituitary extract (BPE), ascorbic acid, and chondroitin sulfate, on cell proliferation, size, and morphology.

**Methods:**

PCEC cultures were seeded at an initial cell density of 400 cells/cm^2^ in the presence or absence of 20,000 murine-irradiated 3T3 fibroblast/cm^2^ in the classic media Dulbecco's Modified Eagle's Medium (DMEM) supplemented with 20% fetal bovine serum (FBS). Mean cell size and bromodeoxyuridine incorporation was assessed at various passages. Growth-promoting factors were studies by seeding PCEC at 8,000 cells/cm^2^ in DMEM with 20% FBS or Opti-MEM I supplemented with 4% FBS and one of the following additives: EGF (0.5, 5, 25 ng/ml), NGF (5, 20, 50 ng/ml), BPE (25, 50, 100, 200 μg/ml), ascorbic acid (10, 20, 40 μg/ml) and chondroitin sulfate (0.03, 0.08, 1.6%), alone or in combination. Cell number, size and morphology of PCEC were assessed on different cell populations. Each experiment was repeated at least twice in three sets. In some cases, cell cultures were maintained after confluence to observe post-confluence changes in cell morphology.

**Results:**

Co-cultures of PCEC grown in DMEM 20% FBS with a 3T3 feeder layer improved the preservation of small polygonal cell shape. EGF, NGF, and chondroitin sulfate did not induce proliferation above basal level nor did these additives help maintain a small size. However, chondroitin sulfate did help preserve a good morphology. BPE and ascorbic acid had dose-dependent effects on proliferation. The combination of BPE, chondroitin sulfate, and ascorbic acid significantly increased cell numbers above those achieved with serum alone. No noticeable changes were observed when PCEC were cocultured with a 3T3 feeder layer in the final selected medium.

**Conclusions:**

Improvements have been made for the culture of PCEC. The final selected medium consistently allowed the growth of a contact-inhibited cell monolayer of small, polygonal-shaped cells.

## Introduction

The corneal endothelium is a single layer of flattened cells adhered to Descemet's membrane. It forms a boundary between the corneal stroma and the anterior chamber. The main role of the corneal endothelium is to maintain corneal transparency by regulating stromal hydration. Since endothelial cell density naturally decreases with age, human corneal endothelial cells (HCEC) are considered to be nonproliferative in vivo. Joyce et al. [1-3] showed that HCEC in vivo are arrested in the G_1_ phase of the cell cycle, while studies by Wilson et al. [4,5] demonstrated that HCEC in vivo retain proliferative capacity.

Previous investigators have developed culture techniques and medium formulation that promote consistent culture of untransformed corneal endothelial cells from human donors [6-11] or animals [12-17]. Effects of numerous growth-promoting agents were tested using cultured HCEC. Samples et al. [18] showed the effect on cell growth of serum, epidermal growth factor (EGF), fibroblasts growth factor (FGF), nerve growth factor (NGF), bovine pituitary extract (BPE), and endothelial cell growth factor. Engelmann et al. [8] improved their previously optimized medium [7] and reduced the serum concentration down to 5% by adding ascorbic acid, insulin, selenium, transferrin, lipids, and FGF. Joyce et al. [10,11] confirmed mitotic or morphologic changes in response to serum, EGF, NGF, and BPE.

Animal models are required for preclinical studies. Because the biology of the eye of the pig is close to that of the human in many respects [19], it would be important to be able to cultivate porcine corneal endothelial cells (PCEC) in a growth medium that allows rapid proliferation while keeping a small cell size with a polygonal morphology at confluence. Several investigators used PCEC cultures for in vitro studies [20-27]. However, few have tested the stimulation of cell proliferation by growth-promoting agents. To our knowledge, only Lee et al. [14] improved the growth of PCEC cultures through their tests of different concentrations of EGF and chondroitin sulfate. Thus, the aim of this study was to further optimize the growth medium used for the culture of PCEC.

The coculture with a feeder layer may help in the initiation of cultures from a low number of cells or from cells with low proliferative ability. The first use of irradiated 3T3 as a feeder layer was reported 30 years ago, when they were shown to favor the formation of cell colonies from diluted human epidermal keratinocytes in culture [28]. This technique led to the clinical application of cultured epidermis for the treatment of burn patients [29,30]. These days, feeder layer of irradiated 3T3 cells are used to coculture many different cell types, including epithelial cells from skin [31] and cornea [32-36]. Alternatively, the growth of cells may be stimulated with additives to culture media. In this study, we evaluated the mitotic changes of PCEC in response to stimulation by growth-promoting agents, such as serum, EGF, NGF, BPE, ascorbic acid, and chondroitin sulfate.

## Methods

### Porcine corneal endothelial cell isolation

The use of animal cells in this study followed the guidelines set by our institution for animal cell research. Porcine eyes were obtained from a local slaughterhouse within 24 h after death. Corneas were dissected out of the globes using a curved scissors (Storz, St. Louis, MO) and washed several times in Dulbecco's Modified Eagle's Medium (DMEM; Invitrogen, Burlington, Ontario, Canada) containing 100 UI/ml penicillin (Sigma, Oakville, Ontario, Canada) and 25 μg/ml gentamicin (Schering Canada, Pointe-Claire, Québec, Canada). Corneas were incubated 30 min at 37 °C in 2.5 mg/ml dispase (Roche, Laval, Québec, Canada). The loosened Descemet's membrane was then peeled off, and endothelial cells were isolated per guidelines described in Zhu et al. [10]. Pooled cells were then centrifuged, resuspended in fresh medium, counted, and plated on dishes coated with fibronectin, collagen, and albumin (FNC; Athena Enzyme Systems, Baltimore, MD) according to the manufacturer's instructions. The FNC coating was used for primary cultures (P0) only and was not used for subsequent passaged cells. Low passaged cells were routinely cryopreserved in 90% FBS (Hyclone, Logan, UT)/10% dimethylsulfoxide (DMSO; Sigma). Unfrozen cells were allowed at least one passage before their use in experiments. After thawing, they exhibited normal cell proliferation and morphology (data not shown). For each study, a Student's t-test was performed to analyze statistical significance with p<0.001 considered to be significant.

### Effect of coculture

**Figure 1:**
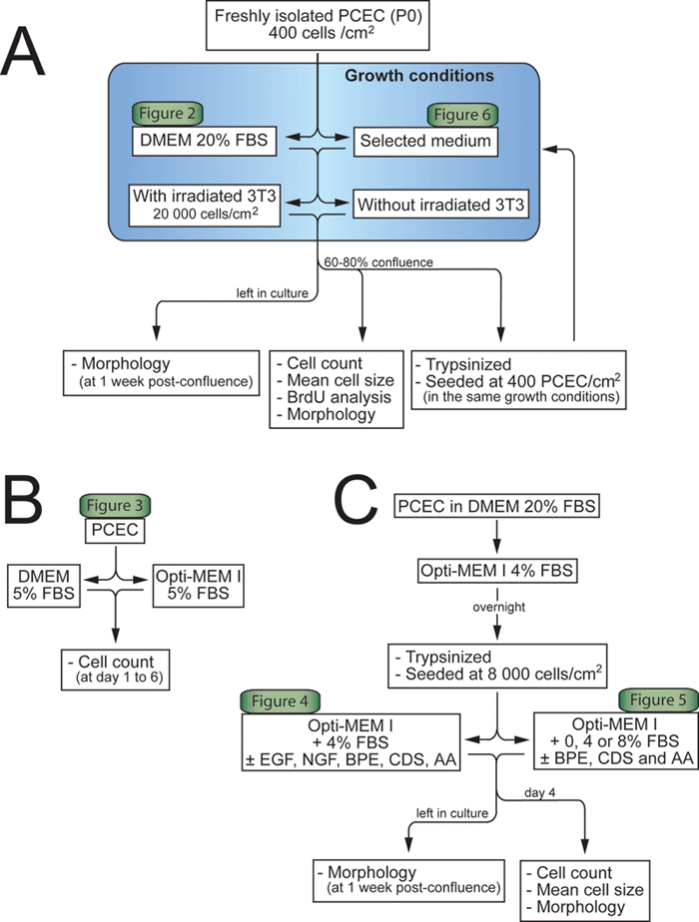
Flow diagram of the experimental protocol. **A**: Effect of coculture. Freshly isolated porcine corneal endothelial cells (PCEC) in primary culture (P0) seeded at 400 cells/cm^2^ were cultured in Dulbecco's Modified Eagle's Medium (DMEM) 20% fetal bovine serum (FBS) or in the selected medium with or without a feeder layer of irradiated murine 3T3 cells. Cells were trypsinized at 60-80% confluence and subcultured in the same conditions. Cell number, mean cell size, and bromodeoxyuridine (BrdU) analysis were assessed on the pre-confluent cells. Other cultures were maintained one week after confluence to document their morphology at post-confluence. **B**: Effect of basal media. PCEC were seeded at 12,000 cells/cm^2^ either in DMEM or Opti-MEM I, both supplemented with 5% FBS. Cells of each well were counted from day 1 to day 6. **C**: Effect of growth promoting additives. PCEC seeded at 8,000 cells/cm^2^ were cultured in DMEM 20% FBS and switched to Opti-MEM I 4% FBS overnight. PCEC were then trypsinized and plated at 8,000 cells/cm^2^ and cultured in the presence of the following additives (used alone or in combination): epidermal growth factor (EGF), nerve growth factor (NGF), bovine pituitary extract (BPE), ascorbic acid (AA), and chondroitin sulfate (CDS). Cell number, size, and morphology were assessed on day 4. Also, the selected medium containing BPE, CDS, and AA was tested in the presence (4% and 8%) or absence of serum. See the methods section for further details.

Freshly isolated PCEC in primary culture (P0) seeded at 400 cells/cm^2^ were cultured in DMEM 20% FBS or in the selected medium in plastic flasks with or without a feeder layer of irradiated murine 3T3 cells (20,000 3T3/cm^2^). Cells were trypsinized at 60-80% confluence and subcultured in the same conditions. Cell number and mean cell size was assessed using a cell counter and particle size analyzer (Beckman Coulter Z2, Mississauga, Ontario, Canada) set to evaluate mean cell size of cells counted between 7.9 and 24.1 μm. Experiments were done three times in triplicate using different pooled cells, and three different counts were done for each sample. Other cultures were maintained one week after confluence to document their morphology at post-confluence. For each condition, three other flasks were used for bromodeoxyuridine (BrdU; Sigma) analysis (Figure 1A).

### Bromodeoxyuridine analysis

The 60-80% confluent cultures were exposed to medium containing 10 μM BrdU for 45 min at 37 °C. The cultures were then rinsed, trypsinized, and the cells fixed in 70% ethanol. Cells were kept at -20 °C until staining. Fixed PCEC were stained with a fluorescein isothiocyanate (FITC)-conjugated mouse anti-BrdU monoclonal antibody (clone 3D4; BD Pharmingen, Oakville, Ontario, Canada) as well as propidium iodide (Sigma) following a modified version of a method developed by Van Erp et al. [37]. Stained cells were analyzed by flow cytometry on a FACScalibur (Becton Dickinson) and analyzed using the program CellQuestPro (Becton Dickinson). For negative control, cells were stained with FITC-conjugated mouse IgG_1κ_ monoclonal isotype control antibody (clone MOPC-21; BD Pharmingen).

### Effect of basal media

PCEC were seeded at 12,000 cells/cm^2^ in six-well plates either in DMEM or Opti-MEM I (Invitrogen), both supplemented with 5% FBS. Triplicate wells were counted twice as previously described from day 1 to day 6 (Figure 1B). Opti-MEM I (Invitrogen) is a modification of Eagles Minimum Essential Medium, buffered with HEPES and sodium bicarbonate (2.4 g/l) and supplemented with hypoxanthine, thymidine, sodium pyruvate, L-glutamine, and trace elements. Calcium chloride (0.2 g/l) was added in the Opti-MEM I solution.

### Effect of growth-promoting additives

PCEC routinely passaged at 8,000 cells/cm^2^ were cultured in DMEM 20% FBS and switched to Opti-MEM I (Invitrogen) supplemented with 0.2 g/l CaCl_2_ and 4% FBS and antibiotics overnight. PCEC were then trypsinized and plated at 8,000 cells/cm^2^ and cultured in the presence of the following additives (used alone or in combination): EGF (0.5, 5, and 25 ng/ml; Austral Biologicals, San Ramon, CA), NGF from mouse submaxillaries (5, 20, 50 ng/ml; Biomedical Technologies, Stoughton, MA), BPE (25, 50, 100, and 200 μg/ml; Biomedical Technologies), ascorbic acid (10, 20, and 40 μg/ml), and chondroitin sulfate A sodium salt from bovine trachea (0.03, 0.08, and 1.6%; Sigma). Cell number, size and morphology were assessed on day 4. Each condition was done at least twice in triplicate using different pooled cells, and each sample was counted three times using a cell counter and particle size analyzer that was set to evaluate mean cell size of cells counted between 7.9 and 24.1 μm. Ascorbic acid and chondroitin sulfate were added fresh at every medium change. Also, the medium containing 50 μg/ml BPE, 20 μg/ml ascorbic acid, and 0.08% chondroitin sulfate was tested in the presence (4% and 8%) or absence of serum as previously described. Cells grown without serum were incubated overnight in 4% serum to allow cell adhesion (Figure 1C).

## Results

### Effect of coculture

**Figure 2:**
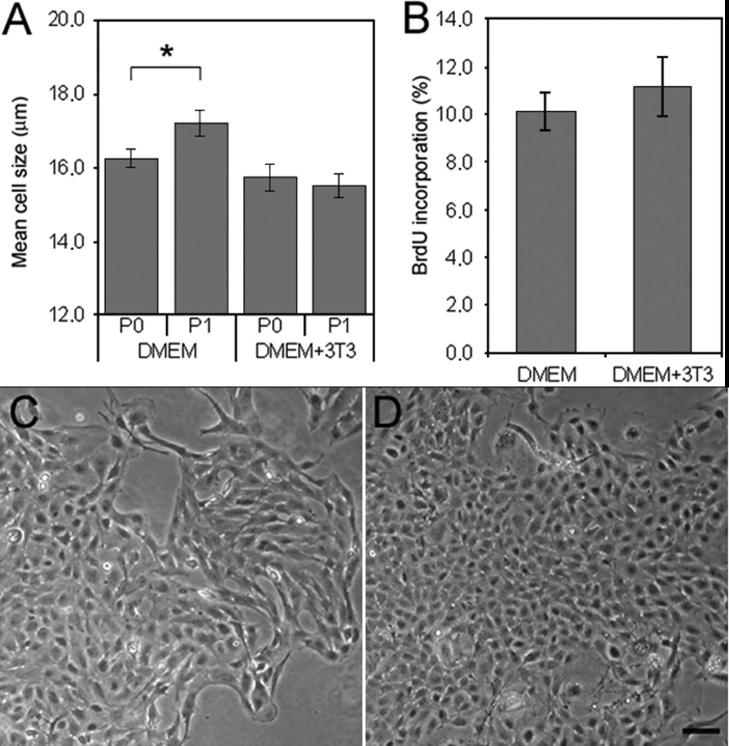
Effect of coculturing porcine corneal endothelial cells (PCEC) with a feeder layer of irradiated 3T3 cells grown in Dulbecco's Modified Eagle's Medium (DMEM) 20% fetal bovine serum (FBS). **A**: Mean cell size of PCEC cultured at different passages (P) after seeding at the very low cell density of 400 cells/cm^2^ without a feeder layer (DMEM) and with a feeder layer (DMEM+3T3). **B**: Percentage of bromodeoxyuridine (BrdU) incorporation of PCEC seeded at 400 cells/cm^2^ at P1 cultured without a feeder layer (DMEM) and with a feeder layer (DMEM+3T3). The results are the mean±SD. Student t-test performed between P0 and P1 (the asterisk indicates a p<0.001). Morphology of PCEC (P1) seeded at 400 cells/cm^2^ and cultured without a feeder layer (**C**) and with a feeder layer (**D**). The scale bar equals 100 μm. Note the smaller size of porcine endothelial cells in the presence of a feeder layer.

We assessed the effect of coculturing PCEC with irradiated 3T3 fibroblasts on mean cell size, morphology, and proliferation using BrdU incorporation. PCEC were cultured in DMEM 20% FBS with or without a feeder layer. Endothelial cells cultured in DMEM 20% FBS with a feeder layer retained their distinctive endothelial morphology in addition to their small size longer than cultures without a feeder layer. At the low seeding density of 400 cells/cm^2^, the cultures at passage 1 (P1) lost their uniformity in size and morphology (Figure 2A,C), whereas cells cultured with a feeder layer at the same density also at P1 retained their small size (Figure 2A) and their typical polygonal morphology (Figure 2D). BrdU analysis demonstrated that cells in both conditions incorporated BrdU at a similar percentage, indicating that the feeder layer did not affect the growth rate (Figure 2B).

### Effect of basal media

**Figure 3:**
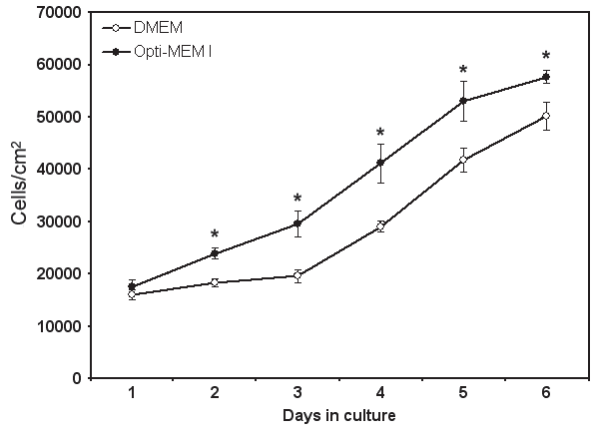
Effect of basal media. Cell counts of PCEC at various days post-seeding. Open circles=cell counts of PCEC grown in Dulbecco's Modified Eagle's Medium (DMEM) supplemented with 5% fetal bovine serum (FBS). Dark circles=cell counts of PCEC grown in Opti-MEM I supplemented with 5% FBS. The results are plotted as the mean±SD. Student t-test performed between DMEM and Opti-MEM I (the asterisk indicates a p<0.001).

We first evaluated the growth response of PCEC cultured with the classic DMEM or with the Opti-MEM I. The same cell populations and the same serum concentration used in both cases permitted a direct comparison of the number of cells after various days in culture. Opti-MEM I induced a statistically significant greater number of cells (after 2, 3, 4, 5, and 6 days) than that of the DMEM (Figure 3). Therefore, Opti-MEM I was used in other experiments for the optimization of the growth medium for PCEC.

### Effect of growth-promoting additives

**Figure 4:**
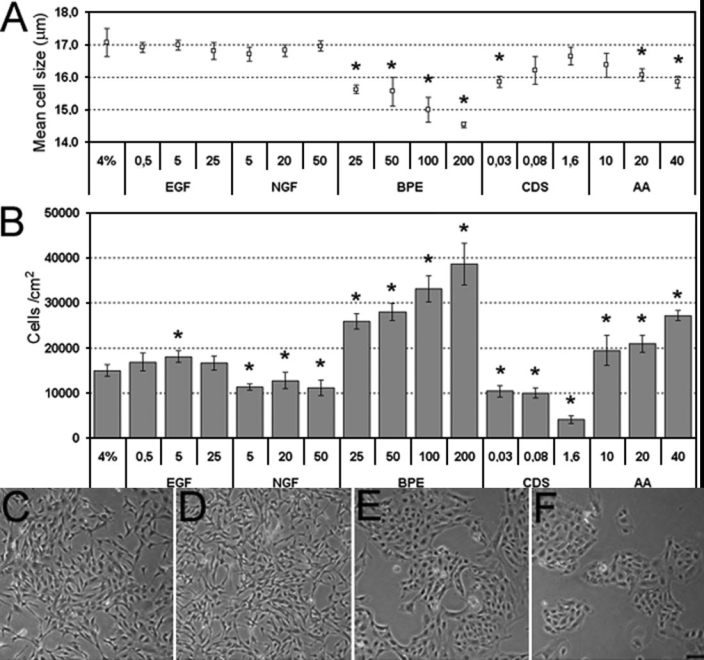
Effect of different additives on cell number, size, and morphology of porcine corneal endothelial cells (PCEC). Representative results of the dose-dependent effect of various growth-promoting factors. PCEC were cultured in Opti-MEM I 4% fetal bovine serum (FBS) and one of the following factors: epidermal growth factor (EGF; 0.5, 5, and 25 ng/ml), nerve growth factor (NGF; 5, 20, and 50 ng/ml), bovine pituitary extract (BPE; 25, 50, 100, and 200 μg/ml), chondroitin sulfate (CDS; 0.03, 0.08, and 1.6%) and ascorbic acid (AA; 10, 20, 40 μg/ml). **A** shows the mean cell size. **B**: Number of cells/cm^2^. The results are the mean±SD. The asterisk indicates a p<0.001 compared to Opti-MEM I 4% FBS (cell number and cell size). Morphology at day 4 of PCEC grown in **C** BPE 50 μg/ml, **D** BPE 200 μg/ml, **E** ascorbic acid 20 μg/ml, and **F** chondroitin sulfate 0.08%. The scale bar is equal to 100 μm.

To study the dose-dependent effect of several growth-promoting factors, PCEC were cultured in a growth medium containing a reduced-serum concentration (Opti-MEM I 4% FBS). The effect of EGF, NGF, BPE, chondroitin sulfate, and ascorbic acid were tested on the proliferative response, morphology, and cell size of PCEC (Figure 4). Figure 4B shows that EGF at concentrations of 0.5 and 25 ng/ml and all concentrations tested for NGF had no significant stimulatory effect on the proliferation of PCEC above basal levels nor did they help maintain a small mean cell size. A concentration of 5 ng/ml had a small but statistically significant increase in cell number, however this concentration did not have a significant effect on mean cell size and morphology. Therefore, EGF and NGF were not kept for the final selected medium.

BPE shows a dose-dependent effect on PCEC growth and mean cell size as shown in Figure 4A and Figure 4B. The highest number and the smallest cells were obtained with a BPE concentration of 200 μg/ml. However, high concentrations of BPE greatly changed the morphology of PCEC. Cells at 200 μg/ml BPE had a fibroblast-like morphology (Figure 4D). In contrast, 50 μg/ml BPE preserved an adequate cell morphology. It was thus chosen as the optimal BPE concentration for PCEC cultures (Figure 4C).

Following the addition of freshly prepared solutions, ascorbic acid had a dose-dependent effect on cell growth as shown in Figure 4B. The highest concentration of ascorbic acid tested (40 μg/ml) yielded the highest cell number and the smallest cells. However, the typical endothelial cell morphology was more closely achieved with 20 μg/ml (Figure 4E).

For PCEC, 0.03 and 0.08% chondroitin sulfate reduced cell growth by, respectively 1.4 and 1.5 times, and 1.6% dramatically reduced (3.6X) the growth of PCEC compared to the control culture (Figure 4B). A concentration of 0.08% chondroitin sulfate was kept in the final selected medium because it was shown to keep cells growing in tightly packed units, thus aiding cell morphology (Figure 4F).

**Figure 5:**
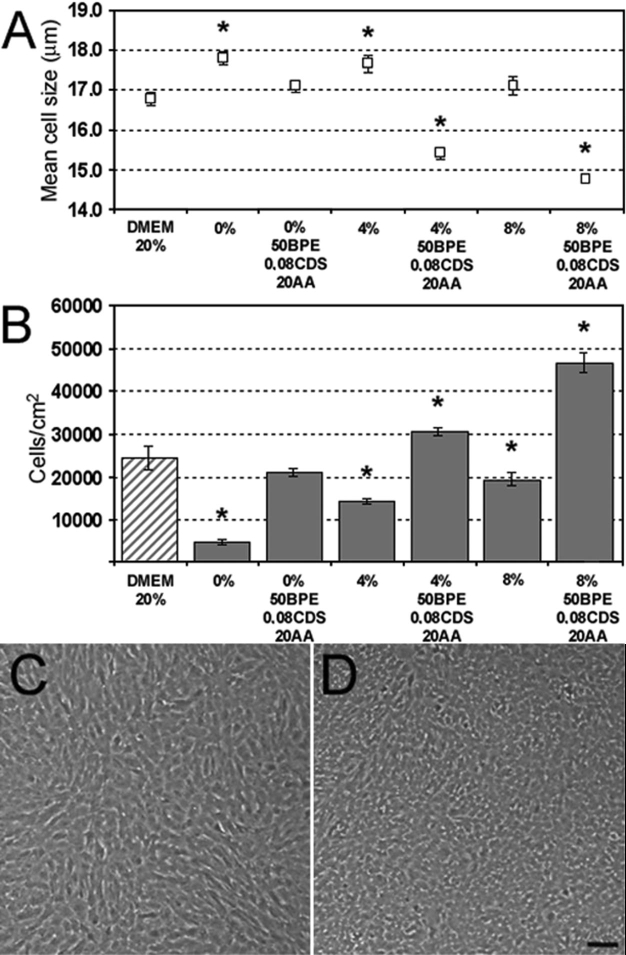
Effect of different culture media formulations on cell number, size, and morphology of porcine corneal endothelial cells (PCEC). Representative results of the additive effect of 50 μg/ml bovine pituitary extract (BPE), 0.08% chondroitin sulfate (0.08 CDS) and 20 μg/ml ascorbic acid (20AA) on (**A**) mean cell size and (**B**) cell number at day 4 on PCEC grown in Opti-MEM I supplemented with 0, 4, or 8% fetal bovine serum (FBS), and compared to the classic Dulbecco's Modified Eagle's Medium (DMEM) 20% FBS (dashed bar graph; mean±SD). The asterisk indicates a p<0.001 compared to DMEM 20% FBS (cell number and cell size). Morphology of PCEC grown in **C** DMEM 20% FBS or **D** the selected medium, consisting of Opti-MEM I supplemented with 8% FBS, 50 μg/ml BPE, 0.08% chondroitin sulfate and 20 μg/ml ascorbic acid. Cells were left in culture one week passed confluence to assess cell morphology of post-confluent cultures. The scale bar is equal to 100 μm. Note that in (**C**) endothelial cells are elongated and of different size whereas in (**D**) they are small and cuboidal cells and have a morphology more characteristic of native cells.

The medium containing the combined factors conducive to the best growth, mean cell size, and morphology was tested (Figure 5) in the presence or absence of serum. Culturing PCEC in Opti-MEM I supplemented with 0%, 4%, or 8% FBS with 50 μg/ml BPE, 20 μg/ml ascorbic acid and 0.08% chondroitin sulfate resulted in a significant increase in cell number when compared to cells grown in Opti-MEM I only (0% FBS) or with serum alone (4% or 8% FBS; Figure 5B). Adding the supplements in 0% serum resulted in a 4.1 fold increase in cell number, and the total cell number in the supplemented 0% serum medium was similar to that achieved with DMEM 20% FBS. When added in 4% and 8% serum, the supplements increased cell number 2.1 and 2.4 times, respectively. Adding the supplements also resulted in a smaller mean cell size. As shown in Figure 5C, PCEC grown in the classic DMEM 20% FBS formed a monolayer of cells harboring different shapes including some that were elongated. However, PCEC grown in our selected medium (8% serum and additives) formed a uniform monolayer of tightly packed cuboidal cells (Figure 5D), indicating that they harbor a morphology similar to the normal endothelial morphology found in vivo.

### Selected medium and 3T3 coculture

**Figure 6:**
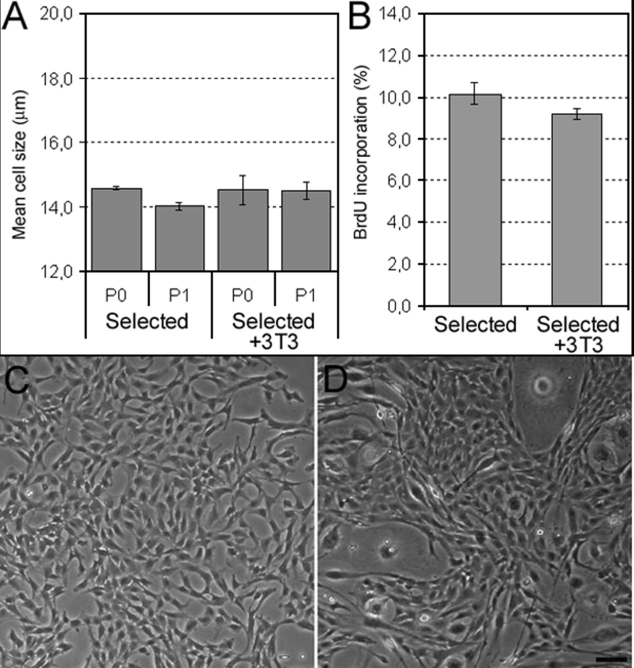
Effect of coculturing porcine corneal endothelial cells (PCEC) with a feeder layer of irradiated 3T3 cells grown in our selected medium. **A**: Mean cell size of PCEC cultured at different passages (P) after seeding at the very low cell density of 400 cells/cm^2^ without a feeder layer (Selected) and with a feeder layer (Selected+3T3). **B**: Percentage of bromodeoxyuridine (BrdU) incorporation of PCEC seeded at 400 cells/cm^2^ at P1 cultured without a feeder layer (Selected) and with a feeder layer (Selected+3T3; mean±SD). The student's t-test performed shows that there is no statistically significant difference between P0 and P1 (p<0.001). Morphology of PCEC (P1) seeded at 400 cells/cm^2^ and cultured without a feeder layer (**C**) and with a feeder layer (**D**), both grown in the selected medium, consisting of Opti-MEM I supplemented with 8% fetal bovine serum (FBS), 50 μg/ml bovine pituitary extract (BPE), 0.08% chondroitin sulfate and 20 μg/ml ascorbic acid. The scale bar is equal to 100 μm.

We then evaluated the effect of coculturing PCEC with a feeder layer of irradiated 3T3 cells grown in this selected medium with a seeding cell density of 400 cells/cm^2^. As shown in Figure 6A, mean cell size at P1 remained small regardless of the presence of 3T3 cells. Morphology was similar (Figure 6C, 6D), as was the percentage of BrdU incorporation (Figure 6B). Thus, no significant effect of the 3T3 feeder layer was found when PCEC were grown in our selected medium.

## Discussion

Since the first cell culture of corneal endothelial cells [38], many investigators have attempted to improve growth culture conditions in order to maximally amplify cells while retaining morphology and function [6-8,10,11,17,18,39]. One way of improving culture conditions is to grow them in coculture with an irradiated feeder layer. The addition of a feeder layer of irradiated fibroblasts supports the formation of colonies from a single cutaneous epithelial cell [28,40]. This powerful method is now applied for the expansion of various cell types for in vitro purposes and clinical applications of cultured epidermis and cornea [29,30,33-36]. Since corneal endothelial cells are in close contact with the corneal stroma through Descemet's membrane, it has been speculated that keratocytes secrete growth factor or nutrients that favor the well being of corneal endothelial cells. When cells are grown in cocultures, fibroblasts are not proliferative (since irradiated) but are still able to secrete nutrients or growth factors. Their beneficial influence is well known for the culture of epithelial cells from skin [28,40] and cornea [32-36]. We have shown that coculturing PCEC with irradiated murine fibroblasts does not affect proliferation. Furthermore, a new media supplemented with serum and additives was identified which allows PCEC to retain their small cell size and morphology on plastic as well as in DMEM 20% FBS in coculture with irradiated fibroblasts.

For cells to grow in vitro, they require a complex mixture of nutrients and growth factors which contributes to an adequate physiological environment. A complete analysis demands well characterized assay systems that allow the discrimination of a single component's contribution to cell growth. In this study, we used direct cell counts to determine the relative effect of various growth-promoting agents on PCEC, as was previously done by many investigators for the optimization of corneal endothelial cell cultures [10,11].

EGF was tested because of its known positive effect on corneal wound healing in ex vivo models and in culture for human [10,11,15,41,42] and porcine [14] cells. Mixed results have been previously reported on the effect of EGF on corneal endothelial cell growth. Some studies show an increase in proliferation for bovine [12,17], rabbit [43], primates [44], and humans [18,45], while others show that EGF either has no effect [30] (human) or has an inhibitory effect [46] (rabbit) on cell growth. EGF can have a different effect, depending on the age of the donor cells. Recent studies using cultured HCEC indicated that EGF has a dose-dependent effect on proliferation in a range of 0.05 to 5 ng/ml, with a peak cell count at 5 ng/ml [10,11]. EGF moderately stimulated proliferation in cells from younger human donors, but did not consistently stimulated proliferation in cells from older donors [10,11]. Using PCEC, a previous study showed that low concentrations of EGF (10 ng/ml) had no significant stimulatory effect while high concentrations (100 ng/ml) stimulated cell growth [14]. Our results are in accordance with this study since the low concentrations of 0.5 to 25 ng/ml did not considerably increase PCEC cell numbers.

NGF is secreted in the aqueous humor [47,48]. Thus, it could influence endothelial cells since immunochemistry studies indicated that HCEC express TrkA [49], the high affinity receptor for NGF [50]. In bovine corneal endothelial cell cultures, concentrations of 1, 10, and 100 ng/ml of NGF failed to significantly stimulate DNA synthesis [17]. A study that used human cells reported that NGF (0.2-200 ng/ml) did not stimulate proliferation, but appeared to have a positive effect on cell morphology [10]. With PCEC, we did not see an effect of this growth factor on proliferation, mean cell size, or morphology.

Of the growth-promoting additives tested in this study, only BPE and ascorbic acid demonstrated a positive effect on cell growth and mean cell size. BPE is a broadly used supplement to culture a variety of epithelial and endothelial cells. It is routinely used as a mitogenic supplement in serum-free growth medium. In addition to its mitogenic activity, BPE contains a variety of growth factors and hormones with reported antioxidant activity [51]. Previous studies have shown a positive effect of BPE on cultured corneal endothelial cells from human origin [10,11]. In this study, the effective concentrations of BPE were between 25-200 μg/ml, which is consistent with a previous study done with HCEC showing that BPE induced a dose-dependent response at concentrations of 0.1 to 100 μg/ml, with a peak cell number reported at 100 μg/ml [10].

Ascorbic acid is an important water-soluble antioxidant found in numerous media. Ascorbic acid (75 μg/ml) added daily to cultured rabbit corneal endothelial cells had an inhibitory effect on cell growth [46]. However, Zhu and Joyce [10,11] routinely included ascorbic acid in their optimized growth medium for HCEC cultures. Ascorbic acid was previously found to have a dose-dependent increase in cell number on human cell cultures at concentrations of 6.25 to 25 μg/ml [8]. Further increases in ascorbic acid concentration apparently reversed this stimulation. A concentration of 20 μg/ml of ascorbic acid maximally stimulated growth [8]. Our results showed a positive effect of 10-40 μg/ml ascorbic acid on the growth of PCEC and are consistent with these previous studies.

Chondroitin sulfate is a mucopolysaccharide with an approximate molecular weight of 50,000 Da. It is found in trace amounts in the human corneal stromal layer [52]. The endothelial protective effect of chondroitin sulfate in the preservation media for cornea in eye banks has been proven by many investigators [53-58], however, reports concerning its effects on corneal endothelial cell cultures are rare. A concentration of 0.08% (0.8 mg/ml) was found to have a positive effect on proliferation and also prevented enlargement of HCEC cultures [6]. Yue et al. [39] showed that low concentrations (100 μg/ml to 1 mg/ml) of chondroitin sulfate had little effect on proliferation. However, high concentrations (13.5 and 25 mg/ml) significantly promoted HCEC growth during the 1- to 2-week incubation period. A study using PCEC demonstrated the addition of 1 mg/ml chondroitin sulfate to the growth medium had no effect on proliferation, and that higher concentrations (25 mg/ml) resulted in inhibition of proliferation [14]. In this study we showed chondroitin sulfate decreased proliferation of PCEC compared to control. However, we kept some chondroitin sulfate in the selected medium because of its beneficial aspect for overall cell morphology.

Culturing PCEC in Opti-MEM I supplemented with 8% FBS, 50 μg/ml BPE, 20 μg/ml ascorbic acid, and 0.08% chondroitin sulfate resulted in a significant increase in cell proliferation when compared to cells grown in Opti-MEM I supplemented with 8% FBS alone (Figure 2B). In confluent cultures, the shape of endothelial cells also provides important information regarding the overall health of the monolayer. Growth in our selected culture medium (Opti-MEM I supplemented with 8% FBS, 50 μg/ml BPE, 20 μg/ml ascorbic acid, and 0.08% chondroitin sulfate) consistently generated cultures of small, polygonal-shaped cells, a morphology closer to that of the endothelium monolayer in vivo. Taken together, our results present species-specific effects of various supplements. For example, 0.5 to 25 ng/ml EGF did not affect proliferation of PCEC, whereas this growth factor has a dose-dependent effect on proliferation of HCEC in a range of 0.05 to 5 ng/ml [10,11]. Such species-specific differences are frequently found in cell cultures of other cell types, such as skin epithelial cells. For instance, high concentrations of calcium inhibits proliferation of mouse epidermal cells [59], but does not affect proliferation of human epidermal cells [30].

In summary, improvements have been made for the culture of PCEC. The present study compared the effect of several growth-promoting agents on proliferation, mean cell size, and morphology of PCEC. EGF, NGF, and chondroitin sulfate did not induce proliferation above basal levels. BPE and ascorbic acid stimulated growth, the combination of which had an additive effect, significantly increasing cell numbers above that achieved with serum alone. This study showed that PCEC cultures can be initiated with a small number of viable cells, such as would be the case from a small biopsy for the culture of autologous cells. The selected medium allowed the production of a contact inhibited cell monolayer that can be grown in sufficient quantities to permit a high cell density seeding of these cells on a stroma for the reconstruction of an autologous posterior cornea in the pig model. Further studies will be required to evaluate the efficiency of the reconstructed corneas to support vision in the pig model following transplantation of these reconstructed porcine corneas before developing clinical applications.
